# Burden of Diabetes and First Evidence for the Utility of HbA1c for Diagnosis and Detection of Diabetes in Urban Black South Africans: The Durban Diabetes Study

**DOI:** 10.1371/journal.pone.0161966

**Published:** 2016-08-25

**Authors:** Thomas R. Hird, Fraser J. Pirie, Tonya M. Esterhuizen, Brian O’Leary, Mark I. McCarthy, Elizabeth H. Young, Manjinder S. Sandhu, Ayesha A. Motala

**Affiliations:** 1 Department of Medicine, University of Cambridge, Cambridge, United Kingdom; 2 Wellcome Trust Sanger Institute, Hinxton, United Kingdom; 3 Department of Diabetes and Endocrinology, Nelson R. Mandela School of Medicine, University of KwaZulu-Natal, Durban, South Africa; 4 Centre for Evidence-Based Health Care, Faculty of Medicine and Health Sciences, Stellenbosch University, Stellenbosch, South Africa; 5 Research and Policy Department, Office of Strategy Management, eThekwini Municipality, Durban, South Africa; 6 Oxford Centre for Diabetes, Endocrinology and Metabolism, University of Oxford, United Kingdom; Medical Clinic, University Hospital Tuebingen, GERMANY

## Abstract

**Objective:**

Glycated haemoglobin (HbA_1c_) is recommended as an additional tool to glucose-based measures (fasting plasma glucose [FPG] and 2-hour plasma glucose [2PG] during oral glucose tolerance test [OGTT]) for the diagnosis of diabetes; however, its use in sub-Saharan African populations is not established. We assessed prevalence estimates and the diagnosis and detection of diabetes based on OGTT, FPG, and HbA_1c_ in an urban black South African population.

**Research Design and Methods:**

We conducted a population-based cross-sectional survey using multistage cluster sampling of adults aged ≥18 years in Durban (eThekwini municipality), KwaZulu-Natal. All participants had a 75-g OGTT and HbA_1c_ measurements. Receiver operating characteristic (ROC) analysis was used to assess the overall diagnostic accuracy of HbA_1c_, using OGTT as the reference, and to determine optimal HbA_1c_ cut-offs.

**Results:**

Among 1190 participants (851 women, 92.6% response rate), the age-standardised prevalence of diabetes was 12.9% based on OGTT, 11.9% based on FPG, and 13.1% based on HbA_1c_. In participants without a previous history of diabetes (n = 1077), using OGTT as the reference, an HbA_1c_ ≥48 mmol/mol (6.5%) detected diabetes with 70.3% sensitivity (95%CI 52.7–87.8) and 98.7% specificity (95%CI 97.9–99.4) (AUC 0.94 [95%CI 0.89–1.00]). Additional analyses suggested the optimal HbA_1c_ cut-off for detection of diabetes in this population was 42 mmol/mol (6.0%) (sensitivity 89.2% [95%CI 78.6–99.8], specificity 92.0% [95%CI: 90.3–93.7]).

**Conclusions:**

In an urban black South African population, we found a high prevalence of diabetes and provide the first evidence for the utility of HbA_1c_ for the diagnosis and detection of diabetes in black Africans in sub-Saharan Africa.

## Introduction

Sub-Saharan Africa (SSA) is experiencing a dramatic increase in diabetes. A consequence of rapid demographic and epidemiological transitions, the number of people with diabetes is projected to more than double to 34.2 million by 2040 [1, 2]. An estimated 66.7% of people living with diabetes in SSA are undiagnosed and therefore more at risk of developing harmful and costly complications, the highest proportion of any region in the world [1]. This poses a huge challenge in many SSA countries where over-burdened and under-resourced health systems already have a shortfall of diabetes services [3, 4].

Consistent and comparable measures of glycaemia are important for accurate screening and diagnosis of diabetes and for population-level surveillance, including inter- and intra-population prevalence comparisons, and subsequent targeting of services and resources to high-risk populations. Glycated haemoglobin (HbA_1c_) is recommended as an additional tool to glucose-based measures (fasting plasma glucose [FPG] and 2-hr plasma glucose (2PG) during an oral glucose tolerance test [OGTT]) for the diagnosis of diabetes [5–7]. However, HbA_1c_ can provide different diabetes prevalence estimates and identifies a different population as having diabetes compared with FPG and OGTT. This degree of discordance varies between populations, by ethnicity, and according to the burden of clinical conditions affecting HbA_1c,_ including anaemias, haemoglobinopathies and infection, potentially limiting the utility of HbA_1c_ for the diagnosis and detection of diabetes [8–10]. However, this has not been established in black sub-Saharan African populations.

Given the potential advantages of using HbA_1c_ for the diagnosis and detection of diabetes in the SSA context [11, 12], evidence on the utility of HbA_1c_ in SSA populations is needed. We therefore assessed the diabetes prevalence estimates, association with established risk factors, and the diagnosis and detection of diabetes based on HbA_1c_, FPG, and OGTT in a black South African population.

## Materials and Methods

### Study design

The Durban Diabetes Study (DDS) was a population-based cross-sectional study of individuals aged >18 years, who were not pregnant, and residing in urban black African communities in Durban (eThekwini municipality) in KwaZulu-Natal (South Africa), conducted between November 2013 and December 2014. A detailed description of the survey design and procedures has been previously published [13]. Written informed consent was obtained from all participants. The DDS was approved by the Biomedical Research Ethics Committee at the University of KwaZulu-Natal (reference: BF030/12) and the UK National Research Ethics Service (reference: 14/WM/1061).

### Data collection

A detailed questionnaire, adapted from the standardised World Health Organization (WHO) STEPwise approach to Surveillance (STEPS) tool, including information on participant health, lifestyle, and socioeconomic indices was administered by trained study personnel [14]. Family history of diabetes was defined as history of diabetes in first-degree relatives. Current smokers were defined as currently smoking any tobacco product even if not daily. Current alcohol users were defined as having consumed any alcoholic beverage in the last month. Physical activity included both work-related and leisure-time activity and included any combination of walking, moderate, or vigorous intensity activities. Low physical activity was defined as doing physical activity on less than five days a week and for less than 600 metabolic equivalents (METs)-min per week. Low fruit and vegetable consumption was defined as fewer than five servings of fruit or vegetables a day [15].

Weight, height, waist circumference, and hip circumference were measured. Three blood pressure readings were obtained with a calibrated automatic electronic device and taken at least five minutes apart by trained study personnel. The mean of the last two readings was used for analysis. Body mass index (BMI) was used as a measure of total body obesity, and waist circumference and waist-to-hip ratio were used as measures of abdominal obesity. Standard WHO criteria were used to define raised blood pressure and obesity [16, 17].

Blood samples were drawn following an overnight fast and were obtained, stored, and tested according to the standard WHO methodology [5, 6]. For the OGTT, venous blood samples were collected, in NaF blood tubes, before and 2 hours after ingestion of 75g glucose monohydrate dissolved in 250 ml water for measurement of plasma glucose. In addition, fasting samples were obtained for HbA_1c_, in EDTA whole blood tubes, and serum lipids, in plain serum tubes. Blood samples were stored in cold boxes maintained at 4–8°C until transported to a laboratory within six hours of collection. Plasma glucose was measured using the glucose oxidase method (ABBOTT ARCHITECT 2: CI 8200, Abbott Laboratories, Chicago, IL, USA). HbA_1c_ was measured using ion-exchange high-performance liquid chromatography (HPLC) (BIORAD VARIANT II TURBO 2.0, Bio-Rad Laboratories, Inc., Hercules, CA, USA), using an instrument certified by the National Glycohaemoglobin Standardization Program (NGSP) and International Federation of Clinical Chemistry and Laboratory Medicine (IFCC). The BIORAD VARIANT II TURBO 2.0 method is not significantly affected by HbS-, HbC-, HbE- and HbD-trait haemoglobin variants [18]. These traits are rare in South African populations; >90% occur in immigrants from other countries whereas >98% of the DDS study population self-reported as Zulu or Xhosa [13, 19, 20]. The inter-assay coefficient of variation for HbA_1c_ was 0.98–2.93% for values of HbA_1c_ between 4.7–10.8%; all were within NGSP acceptable limits. Serum total cholesterol, low-density lipoprotein (LDL) cholesterol, high-density lipoprotein (HDL) cholesterol, and total triglycerides were measured with an autoanalyser (ABBOTT ARCHITECT CI: 6200). Quality control testing was performed at the start and end of each batch or shift. WHO diagnostic criteria for diabetes were used for both glucose-based and HbA_1c_-based measures [5, 6]. History of diabetes and current use of insulin or oral hypoglycaemic drugs were self-reported from the questionnaire. Dyslipidaemia was defined according to the South African guidelines (based on European guidelines) [21].

### Statistical analysis

Statistical analysis was performed in Stata14 software package (StataCorp: College Station, TX, USA). Continuous data are presented as mean with 95% confidence intervals (95% CI) and categorical data as a percentage (95% CI). Age-standardised diabetes prevalences were calculated with the direct method, using the WHO world standard population as the standard. To assess the distribution of risk factors by sex, a χ^2^ test was used for categorical variables and a Student’s t-test for normally distributed continuous variables or the equivalent non-parametric test (Mann–Whitney U test) where the normality assumption was in doubt. We fitted Poisson regression models with a single potential risk factor to obtain crude estimates of association with diabetes. We then used multi-level Poisson regression models with robust standard errors, adjusted for clustering at the household and planning unit cluster level, and for other potential risk factors and confounders, including anaemia and chronic infection, to obtain the adjusted estimates. BMI and waist circumference were included separately in the fully adjusted models as they are highly correlated and likely to be collinear [22]. Risk ratios (RRs) with 95% CI and p values are presented.

Analysis of the sensitivity and specificity of the diabetes definitions was restricted to participants with no history of previous diabetes diagnosis. This is necessary as participants with previous diabetes diagnosis were likely to be on treatment, which is likely to affect HbA_1c_ and glucose measurements. Receiver operating characteristic (ROC) analysis was used to assess the overall diagnostic accuracy of HbA_1c_, using OGTT or FPG as the reference. Youden’s Index (Sensitivity + Specificity –1) was used to determine the optimal HbA_1c_ cut-off for the detection of diabetes using OGTT or FPG as the reference [23].

## Results

Of 1300 individuals invited to join the study, 1204 participated (response rate 92.6%); this analysis includes 1190 subjects (851 women) on whom complete data were available. Table 1 shows the characteristics of the total study group by sex. The mean age was 39.7 years (95% CI 38.8–40.7). Mean BMI, waist circumference, FPG, 2-hour plasma glucose, HbA_1c_, total cholesterol, LDL and prevalence of HIV and low physical activity were higher in women. Mean systolic blood pressure and prevalence of smoking and alcohol use were higher in men.

**Table 1:** Characteristics of the total study population by sex (n = 1190).

Characteristic	Total (N = 1190)	Men (N = 339)	Women (N = 851)
Age (years)	39.7 (38.8–40.7)	36.4 (34.8–38.0)	41.1 (39.9–42.2)
BMI (kg/m^2^)	29.2 (28.7–29.7)	23.3 (22.8–23.8)	31.5 (30.9–32.2)
Waist circumference (cm) *	95.4 (94.3–96.4)	84.9 (83.7–86.0)	99.5 (98.2–100.8)
Hip circumference (cm) *	110.1 (109.2–111.0)	98.2 (97.2–99.1)	114.9 (113.8–116.0)
Waist-to-hip ratio	0.87 (0.86–0.87)	0.87 (0.86–0.87)	0.87 (0.86–0.87)
Systolic BP (mmHg) *	117.5 (116.3–118.7)	120.0 (118.0–122.0)	116.5 (115.0–118.0)
Diastolic BP (mmHg)	77.6 (76.9–78.4)	76.6 (75.2–78.0)	78.0 (77.1–78.9)
FPG (mmol/l) *	5.1 (4.9–5.2)	4.6 (4.5–4.8)	5.2 (5.1–5.4)
2-hour PG (mmol/l) *	6.1 (5.9–6.4)	5.0 (4.7–5.2)	6.6 (6.3–6.9)
HbA_1c_ (%) *	5.7 (5.6–5.8)	5.4 (5.3–5.5)	5.8 (5.7–5.9)
HbA_1c_ (mmol/mol) *	38.8 (38.0–39.6)	35.6 (43.8–36.4)	40.1 (39.0–41.2)
Haemoglobin (g/dl) *	12.9 (12.8–13.0)	14.5 (14.3–14.6)	12.3 (12.2–12.4)
Total Cholesterol (mmol/l) *	4.3 (4.2–4.4)	4.0 (3.8–4.3)	4.4 (4.3–4.5)
Triglycerides (mmol/l)	1.4 (1.2–1.6)	1.1 (1.0–1.2)	1.5 (1.2–1.8)
HDL (mmol/l)	1.3 (1.27–1.3)	1.3 (1.3–1.4)	1.27 (1.25–1.29)
LDL (mmol/l) *	2.3 (2.24–2.33)	2.0 (1.9–2.1)	2.4 (2.3–2.5)
HIV positive (%) *	45.1 (42.3–48.1)	33.9 (29.1–39.2)	47.5 (44.1–50.8)
Family history of diabetes (%)	32.8 (30.2–35.5)	33.0 (28.2–38.3)	32.7 (29.6–35.9)
Current smoker (%) *	19.2 (17.0–21.5)	48.4 (43.1–53.7)	7.5 (5.9–9.4)
Alcohol user (%) *	14.3 (12.4–16.4)	25.7 (21.3–30.6)	9.8 (7.9–11.9)
Low fruit & vegetable diet (%)	81.4 (79.0–83.6)	82.6 (76.9–85.5)	81.3 (78.5–93.9)
Low physical activity (%) *	46.5 (43.7–49.4)	33.1 (28.3–38.4)	51.9 (48.5–55.2)

Data are mean (95% CI) or percentage (95% CI). Comparisons of characteristics between men and women were done using χ^2^ for categorical variables, t-test or Mann–Whitney U test for continuous variables: * = P<0.001 men vs. women. BMI = body mass index. WC = waist circumference. BP = blood pressure. PG = plasma glucose. LDL = low-density lipoprotein cholesterol. HDL = high-density lipoprotein cholesterol.

The age-standardised prevalence of diabetes was 12.9% (95% CI 11.0–14.9) based on OGTT, 11.9% (95% CI 10.2–13.9) based on FPG-alone, and 13.1% (95% CI 11.2–15.2) based on HbA_1c_ (Table 2). Based on OGTT, the prevalence of impaired glucose tolerance was 3.5% (95% CI 2.6–4.7) and the prevalence of impaired fasting glucose was 0.8% (95% CI 0.4–1.4). Diabetes prevalence was higher in women (14.0%, 13.1%, and 14.4%) than in men (8.5%, 7.3%, and 8.5%) for OGTT, FPG, and HbA_1c_, respectively. Peak prevalence was in the oldest age-group (≥65 years) in women (39.3%, 34.8% and 40.5%) and in the 55-64-year age-group in men (29.0%, 25.8% and 29.0%), for OGTT, FPG, and HbA_1c_, respectively (Table 2). In total, 164 participants had diabetes by any definition, of which 31.1% were previously undiagnosed.

**Table 2:** Age-specific and age-standardised prevalence of diabetes based on oral glucose tolerance test (OGTT), fasting plasma glucose (FPG) and HbA_1c_ (n = 1190).

		Category of Glycaemia					
		OGTT			FPG-alone	2h PG-alone	HbA_1c_
		IFG	IGT	Diabetes	Diabetes	Diabetes	Diabetes
Age (years)	N	% (95% CI)	% (95% CI)	% (95% CI)	% (95% CI)	% (95% CI)	% (95% CI)
**All**							
18–24	284	0.4 (0.05–2.5)	0.4 (0.05–2.5)	0.4 (0.05–2.5)	0.4 (0.05–2.5)	0.4 (0.05–2.5)	0.4 (0.05–2.5)
25–34	272	0.4 (0.05–2.6)	0.0 (-)	4.4 (2.5–7.6)	4.4 (2.5–7.6)	4.4 (2.5–7.6)	4.8 (2.8–8.1)
35–44	186	0.5 (0.08–3.7)	3.2 (1.5–7.0)	5.4 (2.9–9.7)	4.3 (2.2–8.4)	3.2 (1.5–7.0)	4.3 (2.2–8.4)
45–54	194	1.0 (0.3–4.1)	5.7 (3.2–10.0)	21.1 (15.9–27.5)	20.1 (15.0–26.4)	15.0 (10.6–20.7)	23.7 (18.2–30.2)
55–64	144	1.4 (0.4–5.4)	8.3 (4.8–14.1)	33.3 (26.1–41.5)	32.6 (25.5–40.7)	29.2 (22.3–37.1)	31.9 (24.8–40.0)
≥65	109	1.8 (0.5–7.1)	9.2 (5.0–16.3)	34.9 (26.1–41.5)	30.3 (22.4–39.6)	30.3 (22.4–39.6)	35.8 (27.3–45.2)
Missing *	1	-	1 (-)	-	-		-
Total crude	1190	0.8 (0.4–1.5)	3.5 (2.5–4.7)	12.6 (10.8–14.6)	11.8 (10.1–13.7)	10.3 (8.7–12.2)	12.9 (11.1–14.9)
Age-standardised		0.8 (0.4–1.4)	3.5 (2.6–4.7)	12.9 (11.0–14.9)	11.9 (10.2–13.9)	10.6 (8.8–12.4)	13.1 (11.2–15.2)
**Men**							
18–24	95	0.0 (-)	0.0 (-)	1.1 (1.5–7.2)	1.1 (0.2–7.2)	1.1 (0.2–7.2)	1.1 (0.2–7.2)
25–34	88	1.1 (0.2–7.8)	0.0 (-)	4.6 (1.7–11.6)	4.6 (1.7–11.6)	4.6 (1.7–11.6)	5.7 (2.4–13.0)
35–44	62	1.6 (0.2–10.8)	1.6 (0.2–10.8)	4.8 (1.6–14.1)	3.2 (0.8–12.2)	3.2 (0.8–12.2)	1.6 (0.2–10.8)
45–54	43	0.0 (-)	7.0 (2.2–19.8)	9.3 (3.5–22.6)	9.3 (3.5–22.6)	7.0 (2.2–19.8)	11.6 (4.9–25.3)
55–64	31	0.0 (-)	3.2 (0.4–20.3)	29.0 (15.6–47.4)	25.8 (13.2–44.1)	25.8 (13.3–44.1)	29.0 (15.6–47.4)
≥65	20	5.0 (0.7–29.4)	20.0 (7.5–43.6)	15.0 (4.8–38.4)	10.0 (2.4–44.1)	15.0 (4.7–38.4)	15.0 (4.7–38.4)
Total crude	339	0.9 (0.3–2.7)	2.7 (1.4–5.0)	7.1 (4.8–10.4)	6.2 (4.1–9.3)	6.5 (4.3–9.7)	7.1 (4.8–10.4)
Age-standardised		1.1 (0.6–1.9)	4.0 (2.9–5.2)	8.5 (7.0–10.2)	7.3 (5.8–8.9)	7.5 (6.4–9.5)	8.5 (7.0–10.2)
**Women**							
18–24	189	0.5 (0.1–3.7)	0.5 (0.1–3.7)	0.0 (-)	0.0 (-)	0.0 (-)	0.0 (-)
25–34	184	0.0 (-)	0.0 (-)	4.4 (2.2–8.5)	4.3 (2.2–8.5)	4.3 (2.3–8.5)	4.4 (2.2–8.5)
35–44	124	0.0 (-)	4.0 (1.7–9.4)	5.7 (2.7–11.4)	4.8 (2.2–10.4)	3.2 (1.2–8.3)	5.7 (2.7–11.4)
45–54	151	1.3 (0.3–5.2)	5.3 (2.7–10.3)	24.5 (18.3–31.0)	23.2 (17.1–30.6)	17.2 (12.0–24.1)	27.2 (20.6–34.8)
55–64	113	1.8 (0.4–6.9)	9.7 (5.5–16.8)	34.5 (26.3–43.8)	34.5 (26.3–43.8)	30.1 (22.3–39.2)	32.7 (24.7–42.0)
≥65	89	1.1 (0.2–7.6)	6.7 (3.0–14.3)	39.3 (29.7–49.9)	34.8 (25.6–45.3)	33.7 (24.6–44.2)	40.5 (30.7–51.0)
Missing *	1	-	1 (-)	-	-	-	-
Total crude	851	0.7 (0.3–1.6)	3.8 (2.7–5.3)	14.8 (12.6–17.4)	14.0 (11.8–16.5)	14.2 (12.0–16.7)	15.2 (12.9–17.7)
Age-standardised		0.7 (0.3–1.3)	3.5 (2.6–4.7)	14.0 (12.1–16.1)	13.1 (11.2–15.2)	11.3 (9.5–13.2)	14.4 (12.4–16.5)

Data are percentage (95% CI). OGTT = oral glucose tolerance test. FPG = fasting plasma glucose. 2h PG = 2 hour post-load plasma glucose. IFG = impaired fasting glucose. IGT = impaired glucose tolerance. OGTT and FPG categories of glycaemia were defined according to the World Health Organization (1998) definitions, HbA_1c_ according to the World Health Organization (2011) definition. Proportions include those classified with IFG, IGT or diabetes by OGTT, FPG, 2h PG-alone or HbA_1c_ and those with a previous history of diabetes. Age-standardised = direct standardisation (WHO world standard population).

*Age unknown for one participant.

For all diabetes definitions, when compared with the non-diabetes group, participants with diabetes were older and had a higher prevalence of total body and abdominal obesity, hypertension, dyslipidaemia, and family history of diabetes (Table 3).

**Table 3:** Prevalence and mean estimates of risk factors in participants diagnosed with diabetes based on oral glucose tolerance test (OGTT), fasting plasma glucose (FPG) and HbA_1c_ (n = 1190).

			Diabetes	
Characteristics	Total (n = 1190)	OGTT (n = 150)	FPG (n = 140)	HbA_1c_ (n = 153)
Age (years)	39.1 (38.8–40.7)	55.6 (53.5–57.7)	55.3 (53.1–57.5)	55.4 (53.4–57.5)
Women (%)	71.5 (68.9–74.0)	84.0 (77.2–89.1)	85.0 (78.1–90.0)	84.3 (77.6–89.3)
BMI (kg/m^2^)	29.2 (28.7–29.7)	34.1 (32.4–35.9)	34.4 (32.5–36.2)	34.6 (32.9–36.3)
BMI ≥25 (%)	60.8 (58.0–63.6)	81.9 (74.8–87.3)	82.7 (75.5–88.2)	84.2 (77.5–89.2)
BMI ≥30 (%)	37.7 (35.0–40.5)	63.8 (55.7–71.1)	63.3 (55.0–70.9)	66.5 (58.5–73.5)
Waist circumference (cm)	95.4 (94.3–96.4)	109.2 (106.2–112.3)	109.7 (106.5–112.9)	110.1 (107.1–113.2)
Abdominal obesity (%)	66.8 (64.0–69.4)	87.9 (81.6–92.3)	88.5 (82.0–92.8)	89.5 (83.5–93.5)
Hip circumference (cm)	110.1 (109.2–111.0)	115.3 (112.4–118.1)	115.4 (112.5–118.3)	115.7 (113.0–118.5)
Waist-to-hip ratio	0.87 (0.86–0.87)	0.95 (0.93–0.97)	0.95 (0.93–0.97)	0.95 (0.94–0.97)
Systolic BP (mmHg)	117.5 (116.3–118.7)	129.5 (125.8–133.2)	129.3 (125.4–133.2)	128.3 (124.8–131.9)
Diastolic BP (mmHg)	77.6 (76.9–78.4)	83.0 (81.1–85.0)	82.9 (80.9–85.0)	82.6 (80.7–84.5)
Hypertension (%)	38.9 (3.2–41.7)	75.8 (68.3–82.1)	76.3 (68.5–82.6)	74.3 (66.8–80.7)
FPG (mmol/l)	5.1 (4.9–5.2)	8.4 (7.7–9.2)	8.6 (7.8–9.4)	8.3 (7.6–9.0)
2-hour PG (mmol/l)	6.1 (5.9–6.4)	13.8 (12.6–15.1)	13.9 (12.6–15.3)	13.6 (12.4–14.9)
HbA_1c_ (%)	5.7 (5.6–5.8)	7.9 (7.5–8.3)	8.0 (7.6–8.5)	8.0 (7.6–8.4)
HbA_1c_ (mmol/mol)	38.8 (38.0–39.6)	62.9 (58.3–67.4)	63.9 (59.1–68.8)	63.4 (59.0–67.8)
Haemoglobin (Hb) (g/dl)	12.9 (12.8–13.0)	12.8 (12.6–13.0)	12.8 (12.5–13.0)	12.8 (12.5–13.0)
TC (mmol/l)	4.3 (4.2–4.4)	4.9 (4.8–5.1)	4.9 (4.8–5.1)	4.9 (4.8–5.1)
Elevated TC (%)	38.5 (35.8–41.3)	65.3 (57.4–75.5)	65.7 (57.4–73.1)	64.7 (56.8–71.9)
Triglycerides (mmol/l)	1.4 (1.2–1.6)	1.8 (1.6–2.0)	1.8 (1.6–2.0)	1.8 (1.6–2.0)
Elevated Triglycerides (%)	17.1 (15.1–19.4)	44.7 (36.9–52.7)	44.3 (36.3–52.6)	44.4 (36.7–52.4)
HDL (mmol/l)	1.3 (1.2–1.3)	1.3 (1.2–1.3)	1.3 (1.2–1.3)	1.3 (1.2–1. 3)
Reduced HDL (%)	29.2 (26.7–31.9)	35.3 (28.1–43.4)	35.0 (27.5–43.3)	36.6 (29.3–44.5)
LDL (mmol/l)	2.3 (2.2–2.3)	2.7 (2.6–2.9)	2.7 (2.6–2.9)	2.8 (2.6–2.9)
Elevated LDL (%)	37.6 (34.9–49.4)	60.7 (52.6–68.2)	61.4 (53.1–69.2)	60.8 (52.8–68.2)
HIV positive (%)	45.1 (42.3–48.1)	26.7 (20.2–34.3)	26.4 (1.8–34.4)	24.2 (18.0–31.6)
Family history of diabetes (%)	32.8 (30.2–35.5)	58.0 (49.9–65.7)	60.7 (52.4–68.5)	58.8 (20.8–66.4)
Current smoker (%)	19.2 (17.0–21. 5)	12.7 (8.2–19.0)	12.1 (7.7–18.7)	11.1 (7.1–17.2)
Alcohol user (%)	14.3 (12.4–16. 4)	10.7 (6.6–16.7)	8.6 (4.9–14.5)	7.8 (4.5–13.3)
Low fruit & vegetable diet (%)	81.4 (79.0–83.6)	86.4 (79.7–91.2)	85.4 (78.1–90.5)	83.1 (76.0–88.4)
Low physical activity (%)	46.5 (43.7–49.4)	57.8 (49.7)	57.7 (49.2–65.7)	56.7 (49.6–64.4)

Data are mean (95% CI) or percentage (95% CI). OGTT = oral glucose tolerance test. FPG = fasting plasma glucose. BMI = body mass index. Abdominal obesity = waist circumference ≥94/80 (men/women). BP = blood pressure. Hypertension = systolic blood pressure ≥140 mmHg and/or diastolic blood pressure ≥90 mmHg. PG = plasma glucose. TC = total cholesterol. Elevated TC = TC ≥4.5 mmol/l. Elevated triglycerides = triglycerides ≥1.7 mmol/l. HDL = high-density lipoprotein. Reduced HDL = HDL<1.0/<1.2 mmol/l (men/women). LDL = low-density lipoprotein. Elevated LDL = LDL ≥2.5 mmol/l.

After adjustment for clustering and other potential risk factors and confounders, in the fully adjusted models; older age, higher waist circumference, higher BMI, and family history of diabetes were independently associated with diabetes, for OGTT, FPG, and HbA_1c_. (Table 4). In the fully adjusted models; sex, blood pressure, haemoglobin, HIV status, lipids, smoking status, alcohol use and physical activity were not significantly associated with diabetes, for OGTT, FPG, or HbA_1c_ (Tables A-C in S1 Table).

**Table 4:** Risk factors associated with diagnosis of diabetes by oral glucose tolerance test (OGTT), fasting plasma glucose (FPG), and HbA_1c_ (n = 1190).

	Crude^a^		Partially Adjusted^b^		Fully Adjusted^c^	
	Univariable RR (95% CI)	p value	Multivariable RR (95% CI)	p value	Multivariable RR (95% CI)	p value
**OGTT**						
Age (years)	1.05 (1.05–1.06)	<0.001	1.05 (1.04–1.06)	<0.001	1.05 (1.03–1.06)	<0.001
Women	2.09 (1.38–3.20)	0.001	1.57 (1.10–2.26)	0.013	1.23 (0.67–2.26)	0.29
BMI (kg/m^2^)	1.04 (1.03–1.05)	<0.001	1.03 (1.02–1.03)	<0.001	1.02 (1.01–1.04)	0.001
Waist circumference (cm)	1.03 (1.03–1.04)	<0.001	1.02 (1.02–1.03)	<0.001	1.02 (1.01–1.03)	0.001
Family history of diabetes	2.83 (2.10–3.83)	<0.001	2.84 (1.68–4.81)	<0.001	2.36 (1.67–3.34)	<0.001
**FPG**						
Age (years)	1.05 (1.04–1.06)	<0.001	1.05 (1.04–1.06)	<0.001	1.04 (1.03–1.06)	<0.001
Women	2.26 (1.44–3.53)	<0.001	1.71 (1.28–2.30)	<0.001	1.34 (0.70–2.55)	0.38
BMI (kg/m^2^)	1.04 (1.03–1.05)	<0.001	1.03 (1.03–1.04)	<0.001	1.01 (0.99–1.04)	0.001
Waist circumference (cm)	1.04 (1.03–1.04)	<0.001	1.03 (1.02–1.03)	<0.001	1.02 (1.01–1.03)	0.001
Family history of diabetes	3.17 (2.31–4.35)	<0.001	3.19 (1.78–5.69)	<0.001	2.69 (1.87–3.87)	<0.001
**HbA**_**1c**_						
Age (years)	1.05 (1.04–1.06)	<0.001	1.05 (1.04–1.06)	<0.001	1.04 (1.03–1.06)	<0.001
Women	2.14 (1.41–3.25)	<0.001	1.62 (1.19–2.21)	0.002	1.14 (0.61–2.11)	0.68
BMI (kg/m^2^)	1.04 (1.03–1.05)	<0.001	1.03 (1.03–1.04)	<0.001	1.02 (1.01–1.04)	0.001
Waist circumference (cm)	1.04 (1.03–1.04)	<0.001	1.03 (1.02–1.03)	<0.001	1.02 (1.01–1.03)	<0.001
Family history of diabetes	2.93 (2.17–3.95)	<0.001	2.93 (1.75–4.91)	<0.001	2.44 (1.73–3.46)	<0.001

RR = Risk Ratio. 95% CI = 95% Confidence Interval. OGTT = oral glucose tolerance test. FPG = fasting plasma glucose. BMI = body mass index. WC = waist circumference.

^a^Crude = univariable poisson regression between risk factor and diabetes.

^b^Partially Adjusted = multivariable poisson regression adjusted for age, sex and clustering at the planning unit cluster and household level.

^c^Fully adjusted = multivariable poisson regression adjusted for age, sex, waist circumference or BMI, blood pressure, haemoglobin, lipids, HIV infection, family history of diabetes, smoking status, alcohol use, physical activity and clustering at the planning unit cluster and household level.

**Fig 1:**
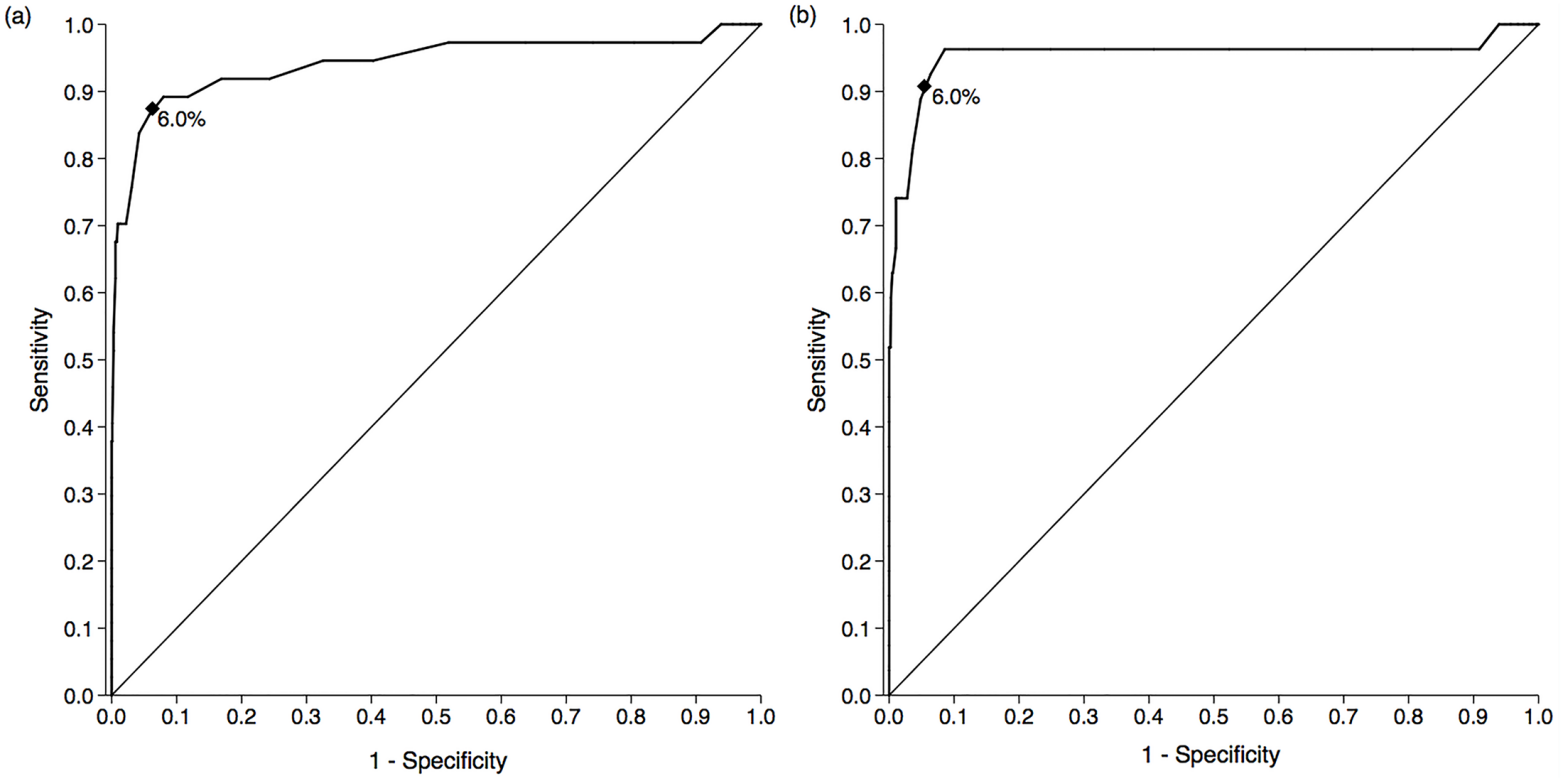
Receiver Operating Curve (ROC) curves for HbA_1c_ for the detection of diabetes with OGTT (a) and FPG (b) as the reference. Area under the ROC curve: 0.94 (95% CI 0.88–0.99) with oral glucose tolerance test (OGTT) as the reference (a) and 0.95 (95% CI 0.88–1.00) with fasting plasma glucose (FPG) as the reference (b). Optimal HbA_1c_ cut off: 42 mmol/mol (6.0%) with OGTT as the reference (sensitivity 89.2%, specificity 92.0%) (a), and 42 mmol/mol (6.0%) with FPG as the reference (sensitivity 96.3%, specificity 91.4%) (b).

Analysis of the sensitivity and specificity of the diabetes definitions was restricted to participants with no history of previous diabetes diagnosis (n = 1077); taking into account multiple testing, there were no important differences in prevalence and mean values of risk factors between this sample and the total study population (S2 Table). Using OGTT as the reference, an HbA_1c_ ≥48 mmol/mol (6.5%) detected diabetes with 70.3% sensitivity (95%CI: 52.7–87.8) and 98.7% specificity (95%CI: 97.9–99.4) (AUC 0.94 [95%CI 0.89–1.00]). Using FPG as the reference, an HbA_1c_ of ≥48 mmol/mol (6.5%) detected diabetes with a sensitivity of 74.1% (95% CI: 54.9–93.3%) and specificity of 98.1% (95% CI: 97.3–98.9) (AUC 0.95 [95% CI: 0.88–1.0]). We found the optimal HbA_1c_ cut-off for detection of diabetes to be 42 mmol/mol (6.0%), using OGTT (sensitivity 89.2% [95%CI 78.6–99.8], specificity 92.0% [90.3–93.7]) or FPG (sensitivity 96.3% [95%CI 89.0–100.0], specificity 91.4% [95%CI 89.7–93.2]) as the reference (Fig 1, S3 Table).

## Discussion

In an urban black South African population, we found a high prevalence of diabetes, and show evidence for the utility of HbA_1c_ for the diagnosis and detection of diabetes. Based on OGTT, FPG, and HbA_1c_, diabetes was independently associated with established risk factors, including age, family history of diabetes and obesity. Our findings highlight the need to evaluate the potential role for HbA_1c_ in screening and diagnosis of diabetes in health services in the region.

The 12.9% prevalence of diabetes in the DDS is amongst the highest reported in SSA at more than triple the current International Diabetes Federation (IDF) prevalence estimate for SSA (3.8%), but is similar to that recently reported in urban black Africans in Cape Town (13.1%) [1, 24]. The prevalence of diabetes in the DDS is more than double that found in a previous study in Durban in 1984 (5.3%), and greater than the increase reported in the Cape Town study [25]. Our study highlights the dramatic increase in the prevalence of diabetes in the past 30 years, and confirms that the diabetes epidemic is well established in urban South African populations. This high prevalence of diabetes is likely to be a result of the increasing burden of established risk factors, including obesity and family history of diabetes, in this population. The prevalence of diabetes was markedly higher in women than in men. This is consistent with previous reports of diabetes prevalence in South Africa; however, in some SSA countries men are consistently found to have a higher prevalence of diabetes [1, 24]. Women in the DDS population had significantly higher levels of risk factors for diabetes, including low physical activity and measures of obesity, compared to men, which is, in part, likely explain this disparity.

To our knowledge, the DDS is the first population-based study in a black sub-Saharan African population to assess the utility of HbA_1c_ for the diagnosis and detection of diabetes. Our findings are broadly consistent with a recent pooled analysis of 96 population-based studies which found HbA_1c_ ≥48 mmol/mol (6.5%) to have consistently high specificity and low-to-moderate sensitivity to detect diabetes (using FPG or OGTT as the reference) in populations across 38 countries [8]. However, the sensitivity of HbA_1c_ for detection of diabetes was markedly higher in the DDS population than the pooled analysis [8]. This may be due to methodological differences in HbA_1c_ testing between studies in the pooled analysis or might be a result of true physiological differences in red blood cell turnover and glucose regulation between populations, affecting the relationship between HbA_1c_ and glucose measures in different populations. The optimum HbA_1c_ cut-off for the detection of diabetes in the DDS (≥42 mmol/mol [6.0%]) was consistent with those suggested in other populations, including prospective studies, most of which reported values lower than 48 mmol/mol (6.5%) [8, 9]. This suggests that lowering the HbA_1c_ threshold would increase sensitivity whilst maintaining high specificity.

Some studies have found sizable differences in diabetes prevalence estimates based on HbA_1c_ and glucose-based definitions in populations of African origin outside of SSA [9, 10]. This has led to concerns about the use of HbA_1c_ for population-level surveillance in these populations due to a lack of comparability with glucose-based prevalence estimates [8, 11]. One study, comparing six populations of different ethnicities, included a black Kenyan population and found the prevalence of diabetes using HbA_1c_ to be less than half that found using OGTT [26]. However, this study used a small (n = 296) selected sample and HbA_1c_ was measured using a point of care test and not the standardised laboratory HPLC method recommended for HbA_1c_-based diagnosis of diabetes [6]. By contrast, we found the prevalence of diabetes to be similar using OGTT, FPG, and HbA_1c_, indicating that prevalence estimates using laboratory based HbA_1c_ are comparable to those of glucose-based measures in this population. This is important for the reliable comparison of diabetes burden and distribution in population-based health surveys and for disease surveillance using multiple measures of glycaemia.

The strengths of our study include the population-based sample with OGTT and HbA_1c_ performed in all individuals, as well as extensive assessment of potential confounding factors. Furthermore, laboratory measures were performed uniformly, including the use of validated NGSP and IFCC certified, laboratory-based HPLC assay for HbA_1c_ measurement. Limitations of this study include the use of glucose-based measures as a reference by which to assess the HbA_1c_ definition; glucose-based measures have considerable intra-individual variability that may lead to random misclassification [9, 27]. No single measure of glycaemia captures the phenotypic complexity of diabetes and the risk of its microvascular and macrovascular complications. As such, diagnosis of diabetes in clinical practice is a sequential analytical process including repeated measurement of one or many measures of glycaemia, depending on each patient’s characteristics. The low proportion of men in the DDS is consistently observed in population-based studies in South Africa and may limit the generalisability of the findings [24, 28]. Likely explanations include high levels of unemployment in the townships sampled leading to men moving away for work (migrant labour system) [13, 29].

HbA_1c_ may have several advantages for use in SSA populations. Unlike FPG and OGTT, HbA_1c_ does not require fasting overnight or immediate laboratory handling and samples can be easily stored and transported [11, 12]. HbA_1c_ also appears to be more strongly associated with risk for macrovascular complications and may have potential utility for combined cardiovascular and diabetes risk assessment [30]. However, this is not established in SSA populations. There is a critical need for prospective studies to assess the relationship between HbA_1c_ and glucose-based measures and risk of diabetes and diabetes complications in SSA populations.

HbA_1c_ is also affected by conditions including haemoglobin variants, anaemia and chronic infection (including HIV and malaria), which may distort HbA_1c_ measurements and estimates of prevalence [11]. These conditions can be broadly divided into those that interfere with HbA_1c_ measurement, such as haemoglobin variants which affect the accuracy of the measurements, and those that affect the interpretation of the HbA_1c_ results, such as anaemia and chronic infection. In South African populations, the prevalence of haemoglobin variants and malaria is low and usually restricted to high risk populations, such as immigrants from other countries, for haemoglobin variants, and in populations near the northern border of the country, for malaria [19, 20, 31]. Other studies have shown that anaemia and HIV can falsely raise or lower the HbA_1c_ measurement [32, 33]. However, in the DDS study population, HIV and anaemia were not independently associated (or inversely associated) with diabetes based on OGTT, FPG or HBA_1c_ definitions. Further studies, including prospective studies and studies in populations with a higher prevalence of haemoglobin variants and malaria, are needed to assess the effects of anaemia, erythrocyte abnormalities, and chronic infection on HbA_1c_ measurement and the utility of HbA_1c_ for the diagnosis of diabetes. Furthermore, evaluation of whether the potential advantages of HbA_1c_ result in earlier diagnosis and improvement in outcomes are needed and, in the context of extremely limited access to NGSP and IFCC certified HbA_1c_ laboratory testing across much of SSA [34, 35], the feasibility and cost-effectiveness of large-scale implementation requires investigation in SSA.

## Supporting Information

S1 Table A. Risk factors associated with diagnosis of diabetes by oral glucose tolerance test (OGTT) (n = 1190). B. Risk factors associated with diagnosis of diabetes by fasting plasma glucose (FPG) (n = 1190). C. Risk factors associated with diagnosis of diabetes by HbA_1c_ (n = 1190). (DOCX)

S2 Table Prevalence and mean estimates of risk factors in participants included in ROC analysis (n = 1077) compared to total study population (n = 1190). (DOCX)

S3 Table Sensitivity and specificity of HbA_1c_ cutoffs for detection of diabetes using oral glucose tolerance test (OGTT) and fasting plasma glucose (FPG) as the reference in participants with no history of previous diabetes diagnosis (n = 1077). (DOCX)

## References

[1] International Diabetes Federation. IDF Diabetes Atlas, 7th edition Brussels, Belgium: International Diabetes Federation, 2015.

[2] Mbanya JC, Motala AA, Sobngwi E, Assah FK, Enoru ST. Diabetes in sub-Saharan Africa. Lancet. 2010;375(9733):2254–66. 10.1016/S0140-6736(10)60550-8 20609971

[3] Atun R, Gale EA. The challenge of diabetes in sub-Saharan Africa. Lancet Diabetes Endocrinol. 2015;3(9):675–7. 10.1016/S2213-8587(15)00236-3 26201978

[4] Beran D. The impact of health systems on diabetes care in low and lower middle income countries. Curr Diab Rep. 2015;15(4):591. 10.1007/s11892-015-0591-8 25721248

[5] World Health Organization. Definition, Diagnosis, and Classification of Diabetes Mellitus and its Complications: Report of a WHO Consultation Part 1. Diagnosis and Classification of Diabetes Mellitus. Geneva: World Health Organization, 1999.

[6] World Health Organization. Use of glycated haemoglobin (HbA1c) in the diagnosis of diabetes mellitus: abbreviated report of a WHO consultation. Geneva: World Health Organization, 2011. 26158184

[7] American Diabetes Association. Diagnosis and classification of diabetes mellitus. Diabetes Care. 2014;37 Suppl 1:S81–90. 10.2337/dc14-S081 24357215

[8] NCD Risk Factor Collaboration (NCD-RisC). Effects of diabetes definition on global surveillance of diabetes prevalence and diagnosis: a pooled analysis of 96 population-based studies with 331 288 participants. The Lancet Diabetes & Endocrinology. 2015;3(8):624–37. 26109024 10.1016/S2213-8587(15)00129-1 PMC4673089

[9] Hare MJ, Shaw JE. Ethnicity Considerations in Diagnosing Glucose Disorders. Curr Diabetes Rev. 2016;12(1):51–7. 25669846 10.2174/1573399811666150211112455

[10] Herman WH, Cohen RM. Racial and ethnic differences in the relationship between HbA1c and blood glucose: implications for the diagnosis of diabetes. J Clin Endocrinol Metab. 2012;97(4):1067–72. 10.1210/jc.2011-1894 22238408 PMC3319188

[11] Bonora E, Tuomilehto J. The pros and cons of diagnosing diabetes with A1C. Diabetes Care. 2011;34 Suppl 2:S184–90. 10.2337/dc11-s216 21525453 PMC3632159

[12] Echouffo-Tcheugui JB, Mayige M, Ogbera AO, Sobngwi E, Kengne AP. Screening for hyperglycemia in the developing world: rationale, challenges and opportunities. Diabetes Res Clin Pract. 2012;98(2):199–208. 10.1016/j.diabres.2012.08.003 22975016

[13] Hird TR, Young EH, Pirie FJ, Riha J, Esterhuizen TM, O'Leary B, et al Study profile: the Durban Diabetes Study (DDS): a platform for chronic disease research. Global Health, Epidemiology and Genomics. 2016;1. 10.1017/gheg.2015.3 PMC5732575 29276614

[14] World Health Organization. The WHO STEPwise approach to noncommunicable disease risk factor surveillance (STEPS). Geneva: World Health Organization. 10.2105/AJPH.2015.302962 PMC4695948 26696288

[15] World Health Organization. Global Strategy on Diet, Physical Activity and Health. Department of Chronic Diseases and Health Promotion, 2008.

[16] World Health Organization. Obesity: preventing and managing the global epidemic Report of a WHO consultation. Geneva: World Health Organization, 2000. 11234459

[17] International Diabetes Federation. The IDF consensus worldwide definition of the metabolic syndrome. Brussels, Belgium: International Diabetes Federation, 2006.

[18] Lin CN, Emery TJ, Little RR, Hanson SE, Rohlfing CL, Jaisson S, et al Effects of hemoglobin C, D, E, and S traits on measurements of HbA1c by six methods. Clin Chim Acta. 2012;413(7–8):819–21. 10.1016/j.cca.2011.12.019 22244931 PMC5068911

[19] Krause A, Wainstein T, Essop FB, Goodyear Q. Testing for haemoglobinopathies in Johannesburg, South Africa: a 30-year review. S Afr Med J. 2013;103(12 Suppl 1):989–93. 10.7196/samj.7255 24300645

[20] Wonkam A, Ponde C, Nicholson N, Fieggen K, Ramessar R, Davidson A. The burden of sickle cell disease in Cape Town. S Afr Med J. 2012;102(9):752–4. 22958698 10.7196/samj.5886

[21] Klug E. South African dyslipidaemia guideline consensus statement. S Afr Med J. 2012;102(3 Pt 2):178–87. 22380916 10.7196/samj.5502

[22] Han TS, Sattar N, Lean M. Assessment of obesity and its clinical implications. BMJ. 2006;333(7570):695–8. 17008674 10.1136/bmj.333.7570.695 PMC1584331

[23] Youden WJ. Index for rating diagnostic tests. Cancer. 1950;3(1):32–5. 15405679 10.1002/1097-0142(1950)3:1<32::aid-cncr2820030106>3.0.co;2-3

[24] Peer N, Steyn K, Lombard C, Lambert EV, Vythilingum B, Levitt NS. Rising diabetes prevalence among urban-dwelling black South Africans. PLoS One. 2012;7(9):e43336 10.1371/journal.pone.0043336 22962583 PMC3433459

[25] Omar MA, Seedat MA, Motala AA, Dyer RB, Becker P. The prevalence of diabetes mellitus and impaired glucose tolerance in a group of urban South African blacks. S Afr Med J. 1993;83(9):641–3. 8310354

[26] Christensen DL, Witte DR, Kaduka L, Jorgensen ME, Borch-Johnsen K, Mohan V, et al Moving to an A1C-based diagnosis of diabetes has a different impact on prevalence in different ethnic groups. Diabetes Care. 2010;33(3):580–2. 10.2337/dc09-1843 20009099 PMC2827511

[27] Rutjes AW, Reitsma JB, Coomarasamy A, Khan KS, Bossuyt PM. Evaluation of diagnostic tests when there is no gold standard. A review of methods. Health Technol Assess. 2007;11(50):iii, ix–51. 18021577 10.3310/hta11500

[28] Motala AA, Esterhuizen T, Gouws E, Pirie FJ, Omar MA. Diabetes and other disorders of glycemia in a rural South African community: prevalence and associated risk factors. Diabetes Care. 2008;31(9):1783–8. 10.2337/dc08-0212 18523142 PMC2518345

[29] Rogan N, Lebani L, Nzimande M. Internal Migration and Poverty in KwaZulu-Natal: Findings from Censuses, Labour Force Surveys and Panel Data. SALDRU, University of Cape Town, Cape Town, South Africa, 2009.

[30] Preiss D, Khunti K, Sattar N. Combined cardiovascular and diabetes risk assessment in primary care. Diabet Med. 2011;28(1):19–22. 21210538 10.1111/j.1464-5491.2010.03157.x

[31] Maharaj R, Raman J, Morris N, Moonasar D, Durrheim DN, Seocharan I, et al Epidemiology of malaria in South Africa: from control to elimination. S Afr Med J. 2013;103(10 Pt 2):779–83. 10.7196/samj.7441 24079633

[32] English E, Idris I, Smith G, Dhatariya K, Kilpatrick ES, John WG. The effect of anaemia and abnormalities of erythrocyte indices on HbA1c analysis: a systematic review. Diabetologia. 2015;58(7):1409–21. 10.1007/s00125-015-3599-3 25994072

[33] Kim PS, Woods C, Georgoff P, Crum D, Rosenberg A, Smith M, et al A1C underestimates glycemia in HIV infection. Diabetes Care. 2009;32(9):1591–3. 10.2337/dc09-0177 19502538 PMC2732167

[34] Sobngwi E, Balde N. Translating evidence into practice: improving access to HbA1c in sub-Saharan Africa. Diabetes Voice. 2011;56(Special Issue 1):36–9.

[35] Camara A, Baldé NM, Sobngwi-Tambekou J, Kengne AP, Diallo MM, Tchatchoua APK, et al Poor glycemic control in type 2 diabetes in the South of the Sahara: The issue of limited access to an HbA1c test. Diabetes Research and Clinical Practice. 2015;108(1):187–92. 10.1016/j.diabres.2014.08.025 25697633

