# Supplementary material for: Burden of Diabetes and First Evidence for the Utility of HbA1c for Diagnosis and Detection of Diabetes in Urban Black South Africans: The Durban Diabetes Study
Source: PLoS One. 2016 Aug 25;11(8):e0161966. doi: 10.1371/journal.pone.0161966 (PMC4999239; doi:10.1371/journal.pone.0161966)
Supplement: S1 Table — (DOCX) [file pone.0161966.s001.docx]

S1 Table A: Risk factors associated with diagnosis of diabetes by oral glucose tolerance test (OGTT) (n=1190)

|  | **Crude^a^**  RR (95% CI) | p value | **Partially Adjusted^b^**  RR (95% CI) | p value | **Fully Adjusted**  **Model 1^c^**  RR (95% CI) | p value | **Fully Adjusted**  **Model 2^d^**  RR (95% CI) | p value |
| --- | --- | --- | --- | --- | --- | --- | --- | --- |
| Age (years) | 1.05 (1.04-1.06) | <0.001 | 1.05 (1.04-1.06) | <0.001 | 1.05 (1.03-1.06) | <0.001 | 1.05 (1.03-1.06) | <0.001 |
| Women | 2.14 (1.41-3.25) | <0.001 | 1.57 (1.10-2.26) | 0.013 | 1.23 (0.67-2.26) | 0.29 | 1.39 (0.80-2.56) | 0.29 |
| BMI (kg/m^2^) | 1.04 (1.03-1.05) | <0.001 | 1.03 (1.02-1.03) | <0.001 | - | - | 1.02 (1.01-1.04) | 0.001 |
| Waist circumference (cm) | 1.04 (1.03-1.04) | <0.001 | 1.02 (1.02-1.03) | <0.001 | 1.02 (1.01-1.03) | 0.001 | - | - |
| Systolic blood pressure (mmHg) | 1.02 (1.01-1.02) | <0.001 | 1.01 (1.00-1.01) | 0.018 | 1.00 (0.99-1.02) | 0.71 | 1.00 (0.99-1.02) | 0.78 |
| Diastolic blood pressure (mmHg) | 1.03 (1.02-1.03) | <0.001 | 1.01 (1.00-1.02) | 0.032 | 1.00 (0.98-1.02) | 0.82 | 1.01 (0.98-1.16) | 0.86 |
| Haemoglobin (g/dl) | 0.95 (0.88-1.02) | 0.17 | 1.08 (0.96-1.21) | 0.23 | 0.99 (0.87-1.13) | 0.91 | 1.01 (0.88-1.16) | 0.86 |
| Total cholesterol (mmol/l) | 1.08 (1.03-1.13) | 0.002 | 1.08 (1.04-1.13) | <0.001 | 1.16 (0.71-3.03) | 0.30 | 1.45 (0.69-3.04) | 0.32 |
| Triglycerides (mmol/l) | 1.01 (1.00-1.03) | 0.072 | 1.02 (0.99-1.04) | 0.18 | 1.16 (0.86-1.56) | 0.33 | 1.21 (0.90-1.63) | 0.20 |
| LDL (mmol/l) | 1.86 (1.62-2.13) | <0.001 | 1.26 (1.08-1.47) | 0.003 | 1.69 (0.30-1.58) | 0.37 | 0.72 (0.31-1.66) | 0.44 |
| HDL (mmol/l) | 0.66 (0.41-1.08) | 0.10 | 0.60 (0.35-1.04) | 0.07 | 0.45 (0.17-1.23) | 0.12 | 0.37 (0.13-1.03) | 0.06 |
| HIV positive | 0.47 (0.33-0.66) | <0.001 | 0.78 (0.53-1.14) | 0.20 | 0.96 (0.62-1.49) | 0.84 | 0.89 (0.57-1.39) | 0.60 |
| Family history of diabetes | 2.93 (2.17-3.95) | <0.001 | 2.84 (1.68-4.81) | <0.001 | 2.36 (1.67-3.34) | <0.001 | 2.45 (1.73-3.48) | <0.001 |
| Current smoker | 0.53 (0.33-0.86) | 0.009 | 1.00 (0.73-1.38) | 0.99 | 1.36 (0.75-2.50) | 0.31 | 1.33 (1.01-1.84) | 0.044 |
| Alcohol user | 0.51 (0.29-0.90) | 0.02 | 1.09 (0.88-1.36) | 0.43 | 1.30 (0.70-2.41) | 0.41 | 1.28 (0.69-2.39) | 0.43 |
| Low fruit and vegetables | 1.12 (0.74-1.69) | 0.58 | 1.41 (0.86-2.33) | 0.18 | 1.59 (0.94-2.70) | 0.09 | 1.58 (0.92-2.70) | 0.10 |
| Low physical activity | 1.50 (1.11-2.03) | 0.008 | 1.06 (0.81-1.39) | 0.66 | 1.15 (0.79-1.65) | 0.47 | 1.21 (0.83-1.75) | 0.33 |

RR=Risk Ratio. 95% CI=95% confidence interval. OGTT=oral glucose tolerance test. FPG=fasting plasma glucose. BMI=body mass index. WC=waist circumference. LDL=low-density lipoprotein. HDL=high-density lipoprotein.

**^a^**Crude=univariable poisson regression between risk factor and diabetes. **^b^**Partially Adjusted=multivariable poisson regression adjusted for age, sex and clustering at the planning unit cluster and household level.

**^c^**Fully adjusted model 1 (no BMI)=multivariable poisson regression adjusted for age, sex, waist circumference, blood pressure, haemoglobin, lipids, HIV infection, family history of diabetes, smoking status, alcohol use, physical activity and clustering at the planning unit cluster and household level

**^d^**Fully adjusted model 2 (no waist circumference)=multivariable poisson regression adjusted for age, sex, BMI (not waist circumference), blood pressure, haemoglobin, lipids, HIV infection, family history of diabetes, smoking status, alcohol use, physical activity and clustering at the planning unit cluster and household level

S1 Table B: Risk factors associated with diagnosis of diabetes by fasting plasma glucose (FPG) (n=1190)

|  | **Crude^a^**  RR (95% CI) | p value | **Partially Adjusted^b^**  RR (95% CI) | p value | **Fully Adjusted**  **Model 1^c^**  RR (95% CI) | p value | **Fully Adjusted**  **Model 2^d^**  RR (95% CI) | p value |
| --- | --- | --- | --- | --- | --- | --- | --- | --- |
| Age (years) | 1.05 (1.04-1.06) | <0.001 | 1.05 (1.04-1.06) | <0.001 | 1.04 (1.03-1.06) | <0.001 | 1.05 (1.03-1.06) | <0.001 |
| Women | 2.26 (1.44-3.53) | <0.001 | 1.71 (1.28-2.30) | <0.001 | 1.34 (0.70-2.55) | 0.38 | 1.53 (0.80-2.93) | 0.20 |
| BMI (kg/m^2^) | 1.04 (1.03-1.05) | <0.001 | 1.03 (1.03-1.04) | <0.001 |  | - | 1.01 (0.99-1.04) | 0.001 |
| Waist circumference (cm) | 1.04 (1.03-1.04) | <0.001 | 1.03 (1.02-1.03) | <0.001 | 1.02 (1.01-1.03) | 0.001 |  | - |
| Systolic blood pressure (mmHg) | 1.02 (1.02-1.03) | <0.001 | 1.00 (1.00-1.01) | 0.05 | 1.00 (0.99-1.02) | 0.61 | 1.00 (0.99-1.02) | 0.67 |
| Diastolic blood pressure (mmHg) | 1.03 (1.02-1.04) | <0.001 | 1.01 (1.00-1.02) | 0.055 | 1.00 (0.98-1.02) | 0.99 | 1 .00 (0.98-1.03) | 0.71 |
| Haemoglobin (g/dl) | 0.95 (0.88-1.03) | 0.21 | 1.08 (0.94-1.24) | 0.27 | 1.01 (0.87-1.16) | 0.93 | 1.03 (0.89-1.19) | 0.70 |
| Total cholesterol (mmol/l) | 1.08 (1.03-1.13) | 0.002 | 1.08 (1.01-1.25) | 0.001 | 1.61 (0.76-3.39) | 0.21 | 1.58 (0.75-3.33) | 0.23 |
| Triglycerides (mmol/l) | 1.01 (1.00-1.03) | 0.075 | 1.02 (0.99-1.04) | 0.21 | 1.12 (0.82-1.53) | 0.48 | 1.18 (0.87-1.61) | 0.28 |
| LDL (mmol/l) | 1.83 (1.58-2.12) | <0.001 | 1.26 (1.10-1.44) | 0.001 | 0.61 (0.26-1.43) | 0.26 | 0.65 (0.28-1.53) | 0.33 |
| HDL (mmol/l) | 0.76 (0.48-1.20) | 0.24 | 0.59 (0.41-0.85) | 0.005 | 0.42 (0.15-1.2) | 0.11 | 0.36 (0.13-0.96) | 0.04 |
| HIV positive | 0.46 (0.33-0.66) | <0.001 | 0.75 (0.52-1.08) | 0.12 | 0.93 (0.59-1.47) | 0.76 | 0.86 (0.54-1.37) | 0.53 |
| Family history of diabetes | 3.17 (2.31-4.35) | <0.001 | 3.19 (1.78-5.69) | <0.001 | 2.69 (1.87-3.87) | <0.001 | 2.80 (1.94-4.03) | <0.001 |
| Current smoker | 0.58 (0.36-0.95) | 0.03 | 0.98 (0.70-1.35) | 0.88 | 1.41 (0.75-2.66) | 0.29 | 1.36 (0.72-2.57) | 0.34 |
| Alcohol user | 0.56 (0.32-0.99) | 0.048 | 0.86 (0.69-1.07) | 0.17 | 1.03 (0.51-2.05) | 0.94 | 0.99 (0.51-1.93) | 0.98 |
| Low fruit and vegetables | 1.33 (0.84-2.12) | 0.22 | 1.30 (0.80-2.11) | 0.29 | 1.47 (0.86-2.53) | 0.16 | 1.44 (0.86-2.44) | 0.17 |
| Low physical activity | 1.56 (1.14-2.15) | 0.005 | 1.19 (0.88-1.30) | 0.52 | 1.14 (0.78-1.68) | 0.49 | 1.20 (0.82-1.75) | 0.34 |

RR=Risk Ratio. 95% CI=95% confidence interval. OGTT=oral glucose tolerance test. FPG=fasting plasma glucose. BMI=body mass index. WC=waist circumference. LDL=low-density lipoprotein. HDL=high-density lipoprotein.

**^a^**Crude=univariable poisson regression between risk factor and diabetes. **^b^**Partially Adjusted=multivariable poisson regression adjusted for age, sex and clustering at the planning unit cluster and household level.

**^c^**Fully adjusted model 1 (no BMI)=multivariable poisson regression adjusted for age, sex, waist circumference, blood pressure, haemoglobin, lipids, HIV infection family history of diabetes, smoking status, alcohol use, physical activity and clustering at the planning unit cluster and household level

**^d^**Fully adjusted model 2 (no waist circumference)=multivariable poisson regression adjusted for age, sex, BMI (not waist circumference), blood pressure, haemoglobin, lipids, HIV infection, family history of diabetes, smoking status, alcohol use, physical activity and clustering at the planning unit cluster and household level

S1 Table C: Risk factors associated with diagnosis of diabetes by HbA_1c_ (n=1190)

|  | **Crude^a^**  RR (95% CI) | p value | **Partially Adjusted^b^**  RR (95% CI) | p value | **Fully Adjusted**  **Model 1^c^**  RR (95% CI) | p value | **Fully Adjusted**  **Model 2^d^**  RR (95% CI) | p value |
| --- | --- | --- | --- | --- | --- | --- | --- | --- |
| Age (years) | 1.05 (1.04-1.06) | <0.001 | 1.05 (1.04-1.06) | <0.001 | 1.04 (1.03-1.06) | <0.001 | 1.04 (1.03-1.06) | <0.001 |
| Women | 2.14 (1.41-3.25) | <0.001 | 1.62 (1.19-2.21) | 0.002 | 1.14 (0.61-2.11) | 0.68 | 1.30 (0.70-2.41) | 0.41 |
| BMI (kg/m^2^) | 1.04 (1.03-1.05) | <0.001 | 1.03 (1.03-1.04) | <0.001 |  | - | 1.02 (1.01-1.04) | 0.001 |
| Waist circumference (cm) | 1.04 (1.03-1.04) | <0.001 | 1.03 (1.02-1.03) | <0.001 | 1.02 (1.01-1.03) | <0.001 |  | - |
| Systolic blood pressure (mmHg) | 1.02 (1.01-1.02) | <0.001 | 1.00 (1.00-1.01) | 0.028 | 1.00 (0.99-1.01) | 0.98 | 1.00 (0.99-1.01) | 0.90 |
| Diastolic blood pressure (mmHg) | 1.03 (1.02-1.03) | <0.001 | 1.01 (1.00-1.02) | 0.036 | 1.00 (0.98-1.02) | 0.74 | 1.01 (0.99-1.03) | 0.50 |
| Haemoglobin (g/dl) | 0.95 (0.88-1.02) | 0.17 | 1.07 (0.92-1.24) | 0.40 | 0.98 (0.85-1.12) | 0.72 | 0.99 (0.87-1.14) | 0.94 |
| Total cholesterol (mmol/l) | 1.08 (1.03-1.13) | 0.002 | 1.08 (1.03-1.13) | 0.001 | 1.42 (0.69-2.90) | 0.34 | 1.40 (0.68-2.88) | 0.36 |
| Triglycerides (mmol/l) | 1.01 (1.00-1.03) | 0.072 | 1.02 (0.99-1.04) | 0.21 | 1.19 (0.89-1.59) | 0.24 | 1.25 (0.94-1.67) | 0.13 |
| LDL (mmol/l) | 1.86 (1.62-2.13) | <0.001 | 1.29 (1.10-1.50) | 0.001 | 0.70 (0.31-1.60) | 0.40 | 0.74 (0.32-1.70) | 0.48 |
| HDL (mmol/l) | 0.66 (0.41-1.08) | 0.10 | 0.48 (0.30-0.79) | 0.004 | 0.46 (0.17-1.22) | 0.12 | 0.37 (0.14-1.01) | 0.05 |
| HIV positive | 0.41 (0.29-0.59) | <0.001 | 0.65 (0.50-0.85) | 0.002 | 0.79 (0.50-1.23) | 0.29 | 0.73 (0.46-1.14) | 0.17 |
| Family history of diabetes | 2.93 (2.17-3.95) | <0.001 | 2.93 (1.75-4.91) | <0.001 | 2.44 (1.73-3.46) | <0.001 | 2.54 (1.79-3.59) | <0.001 |
| Current smoker | 0.53 (0.33-0.86) | 0.009 | 0.85 (0.64-1.12) | 0.25 | 1.27 (0.68-2.38) | 0.45 | 1.24 (0.66-2.31) | 0.50 |
| Alcohol user | 0.51 (0.29-0.90) | 0.02 | 0.78 (0.60-0.99) | 0.042 | 0.94 (0.48-1.86) | 0.87 | 0.93 (0.47-1.83) | 0.84 |
| Low fruit and vegetables | 1.12 (0.74-1.69) | 0.58 | 1.11 (0.75-1.64) | 0.61 | 1.18 (0.73-1.90) | 0.51 | 1.17 (0.72-1.9) | 0.54 |
| Low physical activity | 1.50 (1.11-2.03) | 0.008 | 1.03 (0.81-1.32) | 0.80 | 1.04 (1.03-1.06) | 0.75 | 1.04 (1.03-1.06) | 0.58 |

RR=Risk Ratio. 95% CI=95% confidence interval. OGTT=oral glucose tolerance test. FPG=fasting plasma glucose. BMI=body mass index. WC=waist circumference. LDL=low-density lipoprotein. HDL=high-density lipoprotein.

**^a^**Crude=univariable poisson regression between risk factor and diabetes. **^b^**Partially Adjusted=multivariable poisson regression adjusted for age, sex and clustering at the planning unit cluster and household level.

**^c^**Fully adjusted model 1 (no BMI)=multivariable poisson regression adjusted for age, sex, waist circumference, blood pressure, haemoglobin, lipids, family history of diabetes, smoking status, alcohol use, physical activity and clustering at the planning unit cluster and household level

**^d^**Fully adjusted model 2 (no waist circumference)=multivariable poisson regression adjusted for age, sex, BMI (not waist circumference), blood pressure, haemoglobin, lipids, family history of diabetes, smoking status, alcohol use, physical activity and clustering at the planning unit cluster and household level
