# Supplementary material for: Burden of Diabetes and First Evidence for the Utility of HbA1c for Diagnosis and Detection of Diabetes in Urban Black South Africans: The Durban Diabetes Study
Source: PLoS One. 2016 Aug 25;11(8):e0161966. doi: 10.1371/journal.pone.0161966 (PMC4999239; doi:10.1371/journal.pone.0161966)
Supplement: S2 Table — (DOCX) [file pone.0161966.s002.docx]

S2 Table: Prevalence and mean estimates of risk factors in participants included in ROC analysis (n=1077) compared to total study population (n=1190).

| **Characteristics** | **Total (n=1190)** | **Included in ROC analysis (n=1077)** |
| --- | --- | --- |
| Age (years) * | 39.1 (38.8-40.7) | 38.0 (37.1-39.0) |
| Women (%) | 71.5 (68.9-74.0) | 70.2 (67.4-72.9) |
| BMI (kg/m^2^) | 29.2 (28.7-29.7) | 28.8 (28.2-29.3) |
| BMI ≥25 (%) | 60.8 (58.0-63.6) | 58.8 (55.8-61.7) |
| BMI ≥30 (%) | 37.7 (35.0-40.5) | 35.3 (32.5-38.3) |
| Waist circumference (cm) * | 95.4 (94.3-96.4) | 93.9 (92.9-94.9) |
| Abdominal obesity (%) * | 66.8 (64.0-69.4) | 64.5 (61.6-67.3) |
| Hip circumference (cm) | 110.1 (109.2-111.0) | 109.6 (108.7-110.6) |
| Waist-to-hip ratio | 0.87 (0.86-0.87) | 0.86 (0.85-0.86) |
| Systolic BP (mmHg) | 117.5 (116.3-118.7) | 116.3 (115.1-117.5) |
| Diastolic BP (mmHg) | 77.6 (76.9-78.4) | 77.1 (76.3-77.9) |
| Hypertension (%) * | 38.9 (32.0-41.7) | 34.8 (32.0-37.7) |
| FPG (mmol/l) ** | 5.1 (4.9-5.2) | 4.7 (4.7-4.8) |
| 2-hour PG (mmol/l) ** | 6.1 (5.9-6.4) | 5.4 (5.2-5.5) |
| HbA_1c_ (%) ** | 5.7 (5.6-5.8) | 5.5 (5.4-5.5) |
| HbA_1c_ (mmol/mol) ** | 38.8 (38.0-39.6) | 36.3 (35.8-36.9) |
| Haemoglobin (Hb) (g/dl) | 12.9 (12.8-13.0) | 12.9 (12.8-13.0) |
| TC (mmol/l) * | 4.3 (4.2-4.4) | 4.2 (4.1-4.3) |
| Elevated TC (%) | 38.5 (35.8-41.3) | 35.6 (32.8-38.5) |
| Triglycerides (mmol/l) | 1.4 (1.2-1.6) | 1.4 (1.1-1.6) |
| Elevated Triglycerides (%) | 17.1 (15.1-19.4) | 14.7 (12.7-16.9) |
| HDL (mmol/l) | 1.3 (1.2-1.3) | 1.3 (1.3-1.3) |
| Reduced HDL (%) | 29.2 (26.7-31.9) | 28.9 ((26.2-31.7) |
| LDL (mmol/l) * | 2.3 (2.2-2.3) | 2.2 (2.2-2.3) |
| Elevated LDL (%) | 37.6 (34.9-49.4) | 35.1 (32.3-38.0) |
| HIV positive (%) | 43.6 (40.8-46.5) | 45.4 (42.5-48.4) |
| Family history of diabetes (%) | 32.8 (30.2-35.5) | 29.1 (26.4-31.0) |
| Current smoker (%) | 19.2 (17.0-21.5) | 19.9 (17.6-22.4) |
| Alcohol user (%) | 14.3 (12.4-16. 4) | 14.8 (12.8-17.0) |
| Low fruit & vegetable diet (%) | 81.4 (79.0-83.6) | 81.1 (78.5-83.4) |
| Low physical activity (%) | 46.5 (43.7-49.4) | 45.0 (42.1-48.0) |

Data are mean (95% CI) or percentage (95% CI). Comparisons of characteristics between men and women were done using χ^2^ for categorical variables, t-test or Mann–Whitney U test for continuous variables: *=p<0.05 **= p<0.001 total vs. included in ROC analysis. ROC=receiver operating characteristic analysis OGTT=oral glucose tolerance test. FPG=fasting plasma glucose. BMI=body mass index. Abdominal obesity= waist circumference ≥94/80 (men/women). BP=blood pressure. Hypertension= systolic blood pressure ≥140 mmHg and/or diastolic blood pressure ≥90 mmHg. PG=plasma glucose. TC=total cholesterol. Elevated TC= TC ≥4.5 mmol/l. Elevated triglycerides= triglycerides ≥1.7 mmol/l. HDL=high-density lipoprotein. Reduced HDL= HDL<1.0/<1.2 mmol/l (men/women). LDL=low-density lipoprotein. Elevated LDL= LDL ≥2.5 mmol/l.
