# Supplementary material for: Burden of Diabetes and First Evidence for the Utility of HbA1c for Diagnosis and Detection of Diabetes in Urban Black South Africans: The Durban Diabetes Study
Source: PLoS One. 2016 Aug 25;11(8):e0161966. doi: 10.1371/journal.pone.0161966 (PMC4999239; doi:10.1371/journal.pone.0161966)
Supplement: S3 Table — (DOCX) [file pone.0161966.s003.docx]

S3 Table: Sensitivity and specificity of HbA_1c_ cutoffs for detection of diabetes using oral glucose tolerance test (OGTT) and fasting plasma glucose (FPG) as the reference in participants with no history of previous diabetes diagnosis (n=1077)

| **HbA_1c_ cut-off value (%)** | **Sensitivity**  **% (95% CI)** | **Specificity**  **% (95% CI)** | **Positive predictive value % (95% CI)** | **Negative predictive value % (95% CI)** | **Youden Index** |
| --- | --- | --- | --- | --- | --- |
| **OGTT reference*** |  |  |  |  |  |
| 5.8 | 91.9 (82.7-100.0) | 83.1 (80.6-85.6) | 16.1 (3.8-28.6) | 99.7 (99.3-100.0) | 0.750 |
| 5.9 | 89.2 (78.6-99.8) | 88.3 (86.2-90.4) | 21.3 (7.3-35.3) | 99.6 (99.1-100.0) | 0.775 |
| 6.0† | 89.2 (78.6-99.8) | 92.0 (90.3-93.7) | 28.5 (13.1-43.8) | 99.6 (99.1-100.0) | 0.812 |
| 6.1 | 83.8 (70.8-96.8) | 95.8 (94.5-97.0) | 41.3 (24.0-58.7) | 99.4 (98.9-99.9) | 0.807 |
| 6.3 | 75.7 (59.7-91.6) | 96.9 (95.9-98.0) | 46.7 (28.2-65.1) | 99.1 (98.5-99.6) | 0.726 |
| 6.5‡ | 70.3 (52.7-87.8) | 98.7 (97.9-99.4) | 65.0 (46.7-83.3) | 98.9 (98.3-99.6) | 0.689 |
| 6.7 | 67.6 (49.2-85.9) | 99.4 (99.0-99.9) | 80.7 (65.2-96.1) | 98.9 (98.2-99.5) | 0.668 |
| 7.0 | 54.1 (32.2-75.9) | 99.7 (99.4-100.0) | 87.0 (72.2-100.0) | 98.4 (97.6-99.2) | 0.538 |
| **FPG reference**§ |  |  |  |  |  |
| 5.8 | 96.3 (89.0-100.0) | 82.5 (79.9-85.0) | 12.3 (0.0-25.0) | 99.9 (99.7-100.0) | 0.788 |
| 5.9 | 96.3 (89.0-100.0) | 87.7 (85.5-89. 8) | 16.7 (2.3-31.0) | 99.9 (99.7-100.0) | 0.840 |
| 6.0† | 96.3 (89.0-100.0) | 91.4 (89.7-93.2) | 22.4 (6.4-38.4) | 99.9 (99.7-100.0) | 0.877 |
| 6.1 | 88.9 (76.3-100.0) | 95.0 (93.7-96.4) | 32.0 (13.3-50.7) | 99.7 (99.4-100.0) | 0.862 |
| 6.3 | 81.5 (65.3-97.7) | 96.4 (95.2-97.5) | 36.7 (16.5-56.8) | 99.5 (99.1-99.9) | 0.779 |
| 6.5‡ | 74.1 (54.9-93.3) | 98.1 (97.3-98.9) | 50.0 (28.1-71.9) | 99.3 (98.8-99.8) | 0.721 |
| 6.7 | 74.1 (54.9-93.3) | 99.0 (98.3-99.6) | 62.5 (41.3-83.7) | 99.3 (98.8-99.8) | 0.728 |
| 7.0 | 63.0 (40.0-85.9) | 99.4 (99.0-99.9) | 73.9 (53.0-94.8) | 99.1 (98.5-99.6) | 0.624 |

95% CI=95% Confidence Interval. OGTT=oral glucose tolerance test. FPG=fasting plasma glucose. †≥6.5%= World Health Organizartion 2011 HbA_1c_ diabetes definition cutoff. ‡≥6.0%= Youden Index derived optimum HbA_1c_ cutoff in this population. *area under the reciever operating characteristic curve (AUC) 0.94 (95%CI 0.88-0.99) § AUC 0.95 (95%CI 0.88-1.00)
